# A Qualitative Study on Pre-performance Routines of Diving: Evidence From Elite Chinese Diving Athletes

**DOI:** 10.3389/fpsyg.2020.00193

**Published:** 2020-02-14

**Authors:** Qiang Yao, Feng Xu, Jiabao Lin

**Affiliations:** ^1^Department of Physical Education, Beijing Language and Culture University, Beijing, China; ^2^College of Economics and Management, South China Agricultural University, Guangzhou, China

**Keywords:** Chinese athletes, diving, grounded theory, pre-performance routines, qualitative research

## Abstract

Pre-performance routines (PPRs) are essential motor skills prior to a competition for athletes. But we believe that a specific kind of sport has its own PPRs. Using a qualitative research design, this study conducted in-depth interviews with 14 elite Chinese athletes in competitive diving and their coaches as well as observed and analyzed the behaviors of 13 athletes during diving competitions. The results showed that the divers’ PPRs were constituted of four components (psychological skills, pace setting, mastering competition progress, and behavioral strategies), which could be divided into 20 core categories, with the entire diving process divided into three sections and ten stages, including 13 subcategories in off-platform, 12 subcategories in on-platform and the diving stage. Thus, patterns in the PPRs for divers were established and a clear and comprehensive picture of the diving process, as well as the psychological characteristics and behavioral patterns of athletes during the process, were obtained. The findings entail implications for developing PPRs for diving athletes and future studies on PPRs in other competitive diving and other sports.

## Introduction

Pre-performance routines (PPRs) are pre-arranged sequential thoughts and actions that help athletes stabilize and control their thoughts, emotions, and behaviors prior to a competition (Singer, 2002; Cotterill, 2010). Relevant studies and sports-related research tend to refer to PPRs as a plan, a strategy, a protocol, a technique, and even a ritual (e.g. Cohn et al., 1990; Foster et al., 2006; Lonsdale and Tam, 2008; Mesagno et al., 2019). PPRs involve a variety of psychological skills and behavioral responses, which are widely adopted in different sports; particularly, they are most commonly adopted in the preparation stage of self-paced events involving technical performance (e.g. Lonsdale and Tam, 2008; Clowes and Knowles, 2013). Scholars have suggested that PPRs are extremely important for high-performing or elite athletes; the motor skills of these athletes tend to be over-learned and the operation of the expected movements becomes mechanized, whereby they are executed without conscious attention (Cotterill, 2010). They are thus faced with an increased likelihood of being disturbed by internal and external non-task-related stimuli, which may negatively affect their athletic performance (Beauchamp et al., 1996; Jackson and Baker, 2001; Mesagno et al., 2015). The use of cognitive and behavioral procedures helps these athletes avoid interference and ensures smooth performance in the competition.

Singer (2002) conducted a general analysis of the pre-performance state in self-paced events and concluded that it should include the following steps: (1) self-regulation of thoughts and emotions to a status compatible with the expected movements; (2) narrow, dedicated, and sustained concentration; (3) attainable ideal self-efficacy; (4) appropriate distribution of activated locations in the brain to ensure a quiet mind; (5) maximal focus on the target (frequency and duration) to channel the direction of attention; (6) generating a set of simplified routine behaviors prior to and during the competition; and (7) automatic activation of the above processes to allow effortless, effective, and successful performance in the competition. The ultimate goal is to use the aforementioned steps to create individualized consistent PPRs that can be performed automatically regardless of the situation. The purpose of such routines is to ensure that athletes are continually in their best emotional state with high self-expectations, confidence, and concentration prior to and during the competition. Such self-regulatory techniques are essential as they are the driving force of athletes’ performance during the competition, helping them control and regulate their emotions, thoughts, and attention to remain focused and self-controlled, and to ensure a state of flow (Jackson and Baker, 2001; Cotterill, 2010).

It is evident that athletes who have better motor skills and are able to perform effortlessly during competitions have more advantages than their opponents; however, it is a challenge to ensure effortless performance on a consistent basis during competitions. In order to achieve an optimal performance state, it may be a prerequisite to develop and master a set of effective PPRs. Systematic studies on athletes who could effectively perform PPRs have yielded that, regardless of being a conscious or subconscious behavior, PPRs are associated with motor skills, of which they have become an integral part. To a certain degree, PPRs directly affect athletes’ readiness for their performance and results (Cotterill, 2010; Macnamara et al., 2016). Singer (2002) proposed a general five-step PPR approach applicable to different sports. However, empirical results showed that the proposed model was not specific and detailed enough to provide practical guidance for any given sport. Therefore, it would be theoretically and practically more useful if research could target PPRs with consideration of the characteristics of a specific sport.

The research interest of the present study is competitive diving. Patterns in PPRs in the context of diving have not been thoroughly studied. Since China is one of the leading nations in the sport of diving, this study recruited elite diving athletes and coaches of the Chinese national and provincial teams as research subjects. The qualitative research method was adopted to conduct comprehensive and in-depth investigations, interviews, and observations with the subjects. By analyzing their competition experience and behavioral patterns, a set of PPRs for diving was constructed in order to facilitate relevant studies and improve PPR interventions in the sport of diving.

## Materials and Methods

### Research Subjects

Among the 14 athletes interviewed, 6 were from the national diving team and 8 from a provincial diving team. Among the 8 provincial athletes, 4 had previously been selected for the national team. Among the 4 remaining provincial athletes, 2 had previously received the title of “international professional athlete,” and 2 had been granted the title of “national professional athlete.” The athletes’ mean age was 23 years (min = 15 and max = 39), with experience spanning, on average, 14 years as a professional athlete (min = 7 and max = 22). These athletes had collectively won 18 Olympic gold medals (two of them had won five Olympic gold medals each), 1 Olympic silver medal, and 6 gold medals in diving at the World Aquatics Championships. Three of the athletes had won two Olympic championships (see Table 1).

**TABLE 1:** Basic information of the interviewed athletes.

	Gender		Category		Professional level		# of Olympic gold medals	# of Olympic silver medals	Years of service	
	Male	Female	Platform diving	Springboard diving	International level	National level			<14	≥14
National athlete (*n* = 6)	4	2	2	4	6	0	5	1	1	5
Provincial athlete (*n* = 8)	3	5	2	6	6	2	0	0	7	1

Six coaches (2 male and 4 female) were interviewed, among whom three currently coach in the national team and two had prior experience as a coach for the national team (Table 2).

**TABLE 2:** Basic information of the interviewed coaches.

	Gender		Professional title		Years of service		Best achievement as a coach		
	Male	Female	National coach	Senior coach	<40	≥40	# of Olympic champion coached as the head coach	# of Olympic 1st runner-up coached as the head coach	# of Olympic champion coached as the 1st coach
Coach of the national team (*n* = 3)	2	1	2	1	2	1	2	1	0
Coach of the provincial team (*n* = 3)	0	3	2	1	1	2	1	0	2

### Statistical Analysis Software

The QSR Nvivo 7.0 qualitative analysis software package was used to analyze the data collected during the interviews, while the data collected from the video recordings and observations were analyzed using Noldus’ The Observer 6.0 behavioral analysis software package.

### Data Collection

#### Collection of Interview Data

The purpose of this study was to identify PPRs for diving based on an investigation of Chinese elite diving athletes and their coaches. Based on the research objectives, a semi-structured interview outline was drafted. Next, a team of experts who are providing psychological services to the diving team preparing for the coming Olympic Games (experts, including a professor, a doctoral student, and two postgraduate students of athletic psychology) were invited to examine the coverage and contents of the drafted outline. The final outline was updated based on their suggestions and comments. A Behavioral Event Interview (BEI) was used to identify the characteristics of the skills mastered by the interviewed athletes during competitions. Data related to diving competitions were collected through interviews with the athletes, to determine their behaviors, moods, and psychological strategies at different stages of the diving competitions, as well as through interviews with their corresponding coaches. The interview outlines included items designed to collect the athletes’ BEI data, behaviors during diving competitions, and viewpoints from their coaches.

In addition, a pilot study, based on the drafted outline, was conducted by the present researcher to interview two national team gymnastics athletes to improve the operational skills required for the interviews. The interviews were conducted face-to-face by the present author; the average duration was approximately 30 min, and all interviews were recorded.

#### Collection of Observed Data

The collection of observed data was completed by three people: the first person used a digital video camera to record the behavior of all participating athletes prior to ascending the platform or springboard (off-platform). The second person used a digital video camera to record the behavior of the participating athletes when they were on the platform or springboard (on-platform). The third person was expected to take a video camera and move between the various athletes’ zones (such as coaches’ stand, locker rooms, and warm-up areas) to observe and collect information that was not within the coverage of the other cameras.

### Data Analysis

#### Analysis of the Interview Data

Grounded theory involves the development of a new theory based on raw data, to guide the development of theory driven by qualitative research (Glaser, 2001; Charmaz, 2006). The strategy proposed by grounded theory and the method of constructing theory using qualitative data have been widely applied to different fields and disciplines. Based on the results of comparative analysis, Charmaz (2006) suggested that we should regard grounded theory and corresponding methods as a set of principles and practices, rather than prescriptions or packaged procedures. On that account, a grounded-theory perspective that emphasizes the flexible use of coding guidelines is suitable for addressing the research questions of the present study. Therefore, this theory was employed as the data analysis method, based on the following process: open coding, axial coding, and selective coding were primarily applied; then, key behaviors were identified and a PPR model was developed. The coding system was developed strictly following the qualitative research process. First, two of the present researchers participated in the analysis of all of the collected data independently and established a preliminary coding table according to standard theoretical definitions in psychology. Next, a group discussion was conducted to resolve any disagreement on themes and groupings of behaviors until consensus was reached. Following the construction of the preliminary coding table, the researchers referred to the principles of thematic analysis and combined themes based on their similarities, so as to establish a more complex second-level coding table. Specifically, each theme was compared to each of the other themes, so that any themes with a similar meaning were combined, and themes with contrasting meanings were separated. Following the group discussion, the two researchers consistently agreed on the coding system to be applied.

All records of the interviews were first transcribed by the researcher. Next, the transcripts were checked against the records to ensure the accuracy of the information. Another member of the research team was also invited to conduct a second check of the transcripts to compare them against the records. The verified information was then imported into QSR Nvivo 7.0.

#### Analysis of the Observed Data

The recorded videos were imported into Noldus’ The Observer 6.0. Firstly, the researcher conducted a preliminary coding table for the data, according to the research purpose. Next, a group discussion was conducted to resolve any disagreement on the theme and grouping of the behavior until a consensus was reached.

#### Test for Validity

The five types of validity standards include descriptive validity, interpretive validity, theoretical validity, generalizability, and evaluative validity and triangulation methods were used test the validity of the data.

Firstly, four strategies, used to improve the descriptive validity of the data, were adopted during the data collection process: (1) the interviewer ensured that the recording equipment was functional during the interview; (2) the transcriber ensured that the interviews had been transcribed verbatim; (3) the initial coding (components and categories) were sent to the interviewees for verification, and their suggestions were considered to ensure the accuracy of the terms; (4) finally, both athletes and coaches were interviewed to ensure that the collected data from the two groups complemented one another.

Secondly, two strategies were adopted to improve the interpretive validity of the data: (1) a group of experts, specialized in psychology, was recruited to provide suggestions and guidance throughout the entire research process, from preparation to implementation of the research; (2) during the transcription and coding process, the researcher maintained close communication with the interviewees (athletes and coaches) for advice and suggestions.

Thirdly, expert meetings were organized at each data analysis stage, and the OG-P experts were invited to evaluate the accuracy of the data coding.

Fourthly, the research results were sent to some interviewees, who confirmed that the results accurately reflected their situation and presented some abstract concepts in a more tangible manner, as well as confirming that the results could be internally generalized. This study also received recognition from target readers and relevant professionals, following communication with them; the constructed PPRs achieved acceptable external generalizability.

Fifthly, the researcher comprehensively reviewed and studied existing research and related theories on PPRs, from home and abroad, to gain a full understanding of the technical, psychological, and competitive characteristics of diving. In addition, the researcher also investigated the background of the interviewees in terms of years of service as an athlete or coach, as well as educational background. Expert meetings were held throughout the entire research process to improve its evaluative validity.

Lastly, triangulation, which refers to the use of different data sources, methods, analyses, and theories as a means of ensuring the accuracy of research results, was adopted. In this study, the findings from the interviews were further verified during the observation process, and the findings from the observations were compared with those from the interviews to ensure that the findings from the two methods matched, confirmed, and supplemented one another. Moreover, the study not only included athletes as interviewees but also coaches. During the observation process, both horizontal and vertical comparisons were conducted.

## Results and Analysis

Due to the word count restriction, the original quotes of the interviewees were only included for a limited number of topics.

### Results of the Interviews

The analysis of the interview results with the athletes and coaches yielded four components and 16 categories of PPRs, as presented in Table 3.

**TABLE 3:** List of the components and categories of the PPRs collected through the interviews.

	Interviewees/meaning unit	Athlete (*n* = 14)			Total number of meaning unit	Coach (*n* = 6)			Total number of meaning unit
Component	Category	On-platform	Off-platform	Total		On-platform	Off-platform	Total	
Psychological Skills	Visualizing the moves of the dive	14	7	14	62	6	3	6	10
	Adjusting breathing	14	3	14	23	2	0	2	2
	Self-talk	5	2	5	13	0	0	0	0
	Concentrating	4	0	4	4	2	/	2	2
Pace setting	Pace setting	6	3	6	9	2	2	3	4
Mastering competition progress	Using the other participating athletes as a reference for the progress of the competition	/	8	8	11	/	4	4	4
	Listening to the broadcast and the starting signal prior to beginning their dive	7	/	7	10	1	/	1	1
Behavioral strategies	Warming up	0	14	14	21	0	6	6	11
	Habitual behavior	5	0	5	5	2	0	2	3
	Avoiding watching the dive of other athletes	/	4	4	4	/	2	2	2
	Imitating the moves of the dive	4	10	10	16	2	6	6	8
	Throwing the towel	2	/	2	2	1	/	1	1
	Adjusting the springboard (springboard diving)	5	/	5	5	4	/	4	9
	Looking for the coach	/	14	14	15	/	6	6	6
	Showering	/	9	9	9	/	2	2	2
	Resting	/	14	14	14	/	6	6	10

*Unless specified otherwise, “on-platform” refers to the time period during which the athletes were on the platform or springboard, and “off-platform” refers to the time period during which the athletes were not on the platform or springboard.*

### Results of the Observation

#### Observation Results Off-Platform

The analysis of the behavior of 13 athletes off-platform showed that the athletes tended to exhibit the following behaviors: (1) climbing out of the pool to salute the referee and the audience; (2) listening to or checking the announced score of the dive; (3) showering with hot water and then drying off; (4) returning to the rest zone to rest; (5) warming up; (6) looking for the coach; (7) imitating the moves of the dive; (8) showering; and (9) waiting for their turn of the next dive. In addition, following a comparison between the observed behavior and the results of the interviews, three additional categories related to behavioral strategies emerged as follows: (1) climbing out of the pool to salute the referee and the audience; (2) listening to or checking the announced score of the dive; and (3) showering with hot water and then drying. These three categories were considered supplementary to the findings of the interviews.

#### Observation Results On-Platform

The analysis of the behavior of 13 athletes on-platform showed that the athletes tended to exhibit the following behaviors: (1) physical arousal behavior, such as clenching the fists, treading water, shaking and swinging the legs, bending the knees, rotating the head, and twisting the neck; (2) adjusting the springboard (springboard diving); (3) imitating the moves of the dive (platform diving); (4) throwing the towel; (5) adjusting breathing; (6) listening to the broadcast and the starting signal prior to beginning their dive; (7) habitual behavior, such as rubbing hands, squeezing wrists, touching hips, and rubbing face; (8) standing still; and (9) diving. A comparison of the observed categories with the results of the interview yielded one additional category related to behavioral strategies: standing still.

#### Observation of an Individual Case On-Platform

**FIGURE 1:**
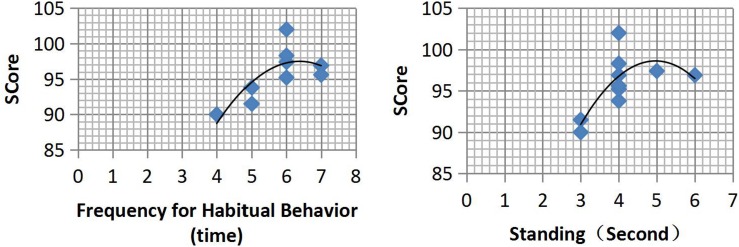
Scatter plots of the relationship between scores and on-platform behaviors of interviewee 5 prior to performing 5253B.

With the development of technological counseling services for the Olympic diving team, and the in-depth investigation of the present study, it was determined that certain associations existed between the behavior of outstanding athletes on the diving platform and their performance during competition. For that reason, the present researchers conducted an investigation on the behaviors of key athletes from the national team during major competitions. The athletes’ habitual behaviors on the diving platform were categorized into movements of the hands, body, and head (including facial expressions). Physical arousal behavior on the platform was divided into upper limb movement and lower limb movement. In addition, habitual behavior that frequently occurred in the same dive group and the order of the movement of each action were analyzed and summarized. Cognitive processes and the stress levels of the athletes accompanying these frequently occurring habitual behaviors were further verified based on observation results and interviews. Since the goal of the present study was not to develop PPRs for a specific athlete or to establish a relationship between PPRs and competition performance, only the data of Athlete 5 (Olympic champion), who performed behavior 5253B in 10 major competitions, were presented and analyzed. The relationship between the frequency of the habitual behaviors, length of time standing still, and achievements in each competition are presented in Table 4 and Figure 1. More data and behavioral analysis information on other athletes are not included in this paper, due to the word limit.

**TABLE 4:** Analysis of the on-platform behaviors of AI5 prior to performing 5253B.

	Scores	On-platform habitual	Standing still
		behavior	(second)
Competition 1	93.8	5	4
Competition 2	96.9	7	4
Competition 3	102	6	4
Competition 4	96.9	7	6
Competition 5	95.2	6	4
Competition 6	91.5	5	3
Competition 7	90	4	3
Competition 8	97.4	6	5
Competition 9	95.6	7	4
Competition 10	98.3	6	4

The frequency of habitual behavior of AI5 ranged between 4 and 7 times and the duration of standing still time ranged between 3 and 6 s prior to performing 5253B. In addition, the resulting score of the performance appeared to be higher when the frequency of the habitual behavior was 6 or 7 times and the duration of the standing still time ranged between 4 and 6 s. A further interview with AI5 showed that the habitual behavior was usually accompanied by self-talk, intense concentration, and adjustment of breathing, while standing still was accompanied by visualizing the moves of the dive.

Analysis of Athlete 5’s behaviors on the diving platform resulted in conclusions regarding their corresponding characteristics and patterns. In addition, different athletes tended to show unique behavioral characteristics and patterns on the diving platform prior to their dives. The characteristics and patterns not only provide the basis for constructing PPRs but also serve as a key reference for coaches and athletes to develop personalized PPRs. It should be emphasized that some of the habitual behaviors on the diving platform were associated with athletes’ psychological skills during the competition. Therefore, it is of great importance to elucidate such a relationship prior to developing PPRs.

### Patterns in the Diving PPRs

The off-platform diving PPRs included the following:

(1) Psychological skills: (a) visualizing the moves of the dive, (b) adjusting breathing, and (c) self-talk; (2) pace setting; (3) mastering competition progress: (a) using the other participating athletes as a reference for the progress of the competition; (4) behavioral strategies: (a) climbing out of the pool to salute the referee and the audience; (b) listening to or checking the announced score of the dive; (c) showering with hot water and then drying the body; (d) returning to the rest zone to rest; (e) standing up and warming up; (f) looking for the coach; (g) imitating the moves of the dive; and (h) showering the body.

The on-platform diving PPRs included the following:

(1) Psychological skills: (a) visualizing the moves of the dive, (b) adjusting breathing, (c) self-talk, and (d) concentrating; (2) pace setting; (3) mastering competition progress: (a) listening to the broadcast and the starting signal prior to beginning their dive; (4) behavioral strategies: (a) physical arousal behavior, (b) adjusting the springboard (springboard diving), (c) imitating the moves of the dive (platform diving), (d) throwing the towel, (f) habitual behavior, and (g) standing still.

**FIGURE 2:**
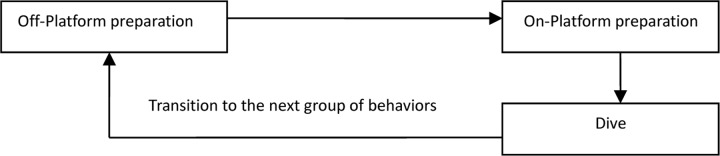
The three diving stages.

**FIGURE 3:**
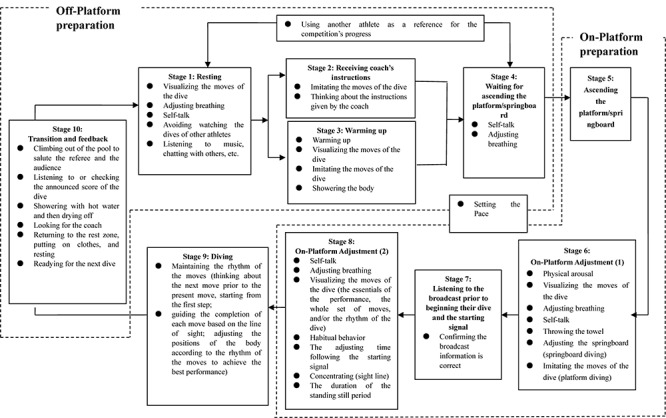
The diving PPRs.

In order to present the distribution of the components and categories of the PPRs during the diving competition more clearly, the diving process was divided into three parts (Figure 2) and 10 stages. Each diving competition consisted of five (women’s competition) or six (men’s competition) such cycles. In light of the findings obtained through the interviews and observations, combined with the three-part model of the diving process, the diving PPRs were constructed (Figure 3).

### Analysis of the Patterns in the Diving PPRs

#### Pace Setting

The category “pace setting” is involved in all parts of the diving PPRs, including the pace of off-platform preparation, the pace of on-stage preparation, and the pace of the dive. Pace setting during the competition is a very important step to ensure effective self-regulation during the competition. One of the interviewees explained “pace setting” as follows:

The behavior of athletes with more experience in competitions lends itself to a certain pace. For example, the usual pace during the Olympic Games is approximately one behavior per minute. The progress of the competitions is usually controlled by the corresponding TV stations, as they need time to broadcast other information, such as advertisements. There is a sense of rhythm in terms of when the athlete should ascend the stairs, when the broadcast announces the scores, when to adjust the springboard, when to throw the towel, when the broadcast announces his/her name, when to step onto the springboard, and when to jump (Coach Interviewee 1).

#### Off-Platform Preparation

##### Stage 1: resting

During this stage, athletes use various strategies of self-regulation, such as visualizing the moves of the dive, adjusting breathing, self-talk, listening to music, and chatting with others, to rest. Other self-regulation strategies include not watching the dives of the other athletes and trying to avoid receiving information related to the performance of other athletes.

##### Stage 2: receiving the coach’s instructions

When the athletes are preparing for the next dive, the coach usually gives instructions related to the essentials of the next dive and the moves that they should pay attention to. The athletes usually reinforce their understanding of the instructions by imitating or visualizing the moves of the dive. It was also found that the sequence of stage 2 and 3 (warm up) depends on the personal habits of the athletes rather than being fixed. Specifically, some of investigated athletes went directly to their coaches for instruction following the rest stage, while other athletes preferred to warm up prior to receiving instructions from their coach.

##### Stage 3: warm up

During this stage, common warm-up activities are carried out; the athletes tend to apply imitation and visualization of the dive moves to activate their body and mind and reinforce the essentials of the dive. In addition, athletes are required to shower and test the water to prepare their body for diving into the water. The time spent on showering and water temperature testing was believed by the interviewees to have a certain impact on the preparation of the dive.

##### Stage 4: waiting to ascend the platform/springboard

At this stage, the athlete waits for the completion of the previous athlete’s dive prior to ascending the platform/springboard. Since the wait is usually not very long, the athlete is required to be alert and aware of when the previous athlete has completed the dive and he/she is able to ascend the platform/springboard. Therefore, the athletes tend to be nervous during this stage and usually adopt self-talk and breathing regulation to assist self-regulation.

##### Stage 10: transition and feedback following the dive

This stage is a transition stage between two dives. A certain number of behaviors is expected from the athletes, such as climbing out of the pool to salute the referee and the audience, listening to or checking the announced score of the dive, showering with hot water and then drying the body, looking for the coach, returning to the rest zone, putting on clothes, and resting. One other important aspect at this stage is to forget the result of the previous dive, regardless of it being good or bad, and swiftly adjust oneself for the next dive. One of the interviewees described this stage as follows:

Regardless of the result of the previous dive, [I] try not to think about it, but rather [I] clear my mind and try to calm down. Usually [my] heart beats fast during this time period, [so I] go and find a place to sit and rest (Athlete Interviewee 2).

#### On-Platform Preparation

##### Stage 5: ascending the platform/springboard

This stage is a transition phase between off-platform and on-platform preparation. The ascent is usually too brief to allow for any other moves to be executed.

##### Stage 6: on-platform adjustment (1)

Athletes’ appearance on the platform/springboard is similar to actors’ appearance on the stage of theaters. Moving from “backstage” to being in front of the audience is likely to generate feelings of nervousness. Young and inexperienced athletes are likely to feel dizziness. In order to avoid potential negative effects of such pressure, athletes usually adopt strategies such as getting physically aroused, imitating the moves of the dive, and adjusting their breathing to alleviate the tension and ready themselves for the dive.

##### Stage 7: listening to the broadcast and the starting signal

At this stage, the athlete is expected to check the accuracy of the broadcast and the information displayed on the big screen. If the information does not match his/her personal information, he/she is supposed to raise his/her hand and ask for a correction. If no errors are identified in the broadcast and on the big screen, he/she is supposed to begin the dive after receiving the starting signal.

##### Stage 8: on-platform adjustment (2)

Although this stage lasts less than 1 min, it greatly affects the athletes’ performance. The results showed that athletes tended to adopt self-regulation strategies such as self-talk, visualizing the moves of the dive, adjusting breathing, and concentrating. Habitual behaviors also occur during this stage and are usually accompanied by self-talk, concentrating, and adjustment of breathing, while standing still is usually accompanied by visualizing the moves of the dive.

##### Stage 9: diving

Very few interviewees mentioned self-regulation during this stage. In addition, the self-regulation strategies described by them appeared to be more abstract and highly personalized. Some examples of the responses are as follows:

[I] think about the essentials of a move prior to executing it. [I] find a good rhythm for the moves, such as [counting] “1, 2, 3” and complete the dive following my own pace. When [I] feel that a move was not executed smoothly, [I] try to think of ways to save it (Athlete Interviewee 8).

[I] begin to think about the essentials of the moves after I take the first step, because that’s when you develop the sense for the dive and think about what should be done (Athlete Interviewee 2).

Once I stand there, the moves become a conditioned reflex in my mind. Once I jump, my mind automatically recalls which move/position is the most important to the dive and how I can optimize my performance. Maybe it’s because I have been a diver for a long time that all the moves have become conditioned reflexes to me (Athlete Interviewee 4).

## Discussion

### Methodology

The advantage of qualitative research lies in the fact that it provides a profound, vivid, and comprehensive description of an object or phenomenon. The distinguishing characteristic of grounded theory is that researchers generally have no theoretical hypothesis prior to the research, but rather establish the theory using a bottom-up approach, by compiling and summarizing the concepts and propositions from the original data. The purpose of this study was to elucidate the PPRs of diving. It was expected that the findings would objectively present the experience of senior diving athletes in China and their existing PPRs and patterns so as to construct a PPR model for athletic diving. The choice of research methods in research generally depends on the research question and objectives. Considering the research questions and objectives of this study, the traditional quantitative research paradigm did not appear to fit the needs of the study. Therefore, the researcher chose the qualitative approach, combining interviews and observations (behavior analysis), to achieve the research objectives.

Nevertheless, there were certain limitations to the methodology of the study. Firstly, since this study mainly focused on the psychological training, behavioral strategies, and behavioral cues prior to and during past diving competitions, the responses of the interviewees were based on memory; hence, certain deviations from reality may have existed. Secondly, following the interviews, the researcher found that some of the concepts and responses required supplementary information for further clarification. Multiple rounds of interviews could have resolved the problem; however, due to several reasons, only a few of the interviewees were re-interviewed in this study. Thirdly, due to restriction of resources, the researcher was not able to conduct on-spot observations and timely interviews for each competition. The majority of the behaviors were recorded by video cameras and analyzed following the competition. Information that was not covered by the cameras was not included, which hindered in-depth revelations of the athletes’ behaviors. Fourthly, only four interviewees were platform diving athletes (two males and two females). This was due to the fact that the majority of the platform-diving athletes were much younger, rendering it difficult to conduct in-depth interviews.

### Research Content

Based on the five-step PPRs and related theory, as proposed by Singer et al., combined with the characteristics of the competitive sport of diving and diving athletes, this study divided the diving process into 10 stages and constructed a model of PPRs of diving. The off-platform preparation (stages 1, 2, 3, 4, and 10) in this study corresponded to the stages of “evaluating,” “feedback,” and “readying” of the five-step routines; the on-platform preparation (stages 5, 6, 7, and 8) corresponded to the stages of “imaging” and “focusing”; while diving (stage 9) corresponded to the stage of “starting the competition with a peaceful mind.” In addition, this study also uncovered the behaviors, mental tactics, and patterns of top athletes in China during different stages of competition and proposed the concept of “pace-setting” of the competition, which was a dominant factor throughout the entire PPRs. Maintaining an appropriate rhythm for each movement during off-platform preparation, on-platform preparation, and the diving process was found to be a key measure in ensuring effective self-control and smooth performance during the competition itself. The model proposed in this study clearly and comprehensively revealed the diving process and the psychological and behavioral patterns of top diving athletes during the competition process, so that developers of PPRs, and other athletes, could accurately grasp the requirements of the competition process. However, in reality, the sequence and connection between some stages are not fixed, and athletes are required to adjust their preparation sequence according to their personal habits. Moreover, when applying the model to design PPRs for diving athletes, the specific behavioral procedures should be determined with consideration of the individual characteristics of each athlete. Training on psychological skills, such as how to adjust breathing, self-talk, visualizing the moves of the dive, and concentrating, should be provided to athletes on a regular basis to improve their ability to use the skills; the athletes should also frequently test and modify the skills with practical application.

Developing personalized PPRs that can be easily mastered by athletes is a difficult task. Each athlete has their own unique characteristics. In order to uncover the indicators and habitual behaviors suitable for each athlete, it is necessary to conduct in-depth interviews, repeated observation of large quantities of video recordings, and careful observation of the training and competition process, followed by further verification. In addition to collecting responses and viewpoints from the head coach, team coach, teammates, and athletes, it is also necessary to conduct research and analysis into the personality and characteristics of individual athletes. Furthermore, in the process of designing PPRs for diving athletes, it is also necessary to regularly conduct psychological skills training for athletes, such breathing control, use of self-talk, visualization of the moves of the dive, and concentration, so as to raise their ability to use these critical skills, and to continuously test and revise these skills during application.

The findings of the study also showed that some habitual behaviors (such as hand movements and hand-body movements) during the on-platform stage were accompanied by psychological skills (such as self-talk, concentrating, and adjustment of breathing), while standing still and looking forward and downward were accompanied by visualizing the moves of the dive. Uncovering the relationship between such behaviors and psychological skills is very important for the formulation of PPRs. It is likely that the PPRs of individual dives and synchronized group dives share some common characteristics as well as distinctive stages.

The present research mainly focused on the PPRs of individual dives; hence, the constructed model is suitable for individual diving athletes in the divisions of platform and springboard diving. However, the study did not collect or analyze data related to two-person synchronized dive teams. Since the PPR routines of synchronized diving are also important to the development of the sport, it is suggested that future studies include subjects from this area of the sport.

## Conclusion

Based on the five-step PPRs and related theory proposed by Singer et al., combined with the characteristics of the competitive sport of diving and diving athletes, this study divided the diving process into 10 stages and constructed a model for diving PPRs. The model revealed the behaviors and psychological strategies and patterns of top diving athletes in China during various stages of the competition. In addition, the concept of competition “pace-setting” was proposed.

Off-platform preparation consisted of 13 categories, including: (1) visualizing the moves of the dive; (2) adjusting breathing; (3) self-talk; (4) pace setting; (5) tracking progress using other athletes as a reference; (6) climbing out of the pool to salute the referee and the audience; (7) listening to or checking the announced score of the dive; (8) showering with hot water and then drying off; (9) returning to the rest zone to rest; (10) standing up and warming up; (11) imitating the moves of the dive; (12) looking for the coach; and (13) showering.

On-platform preparation consisted of 12 categories, including: (1) visualizing the moves of the dive; (2) adjusting breathing; (3) self-talk; (4) concentrating; (5) pace setting; (6) listening to the broadcast and starting signal prior to the dive; (7) physical arousal behavior; (8) adjusting the springboard (springboard diving); (9) imitating the moves of the dive (platform diving); (10) throwing the towel; (11) habitual behavior; and (12) standing still.

The third part is diving. The three parts are connected to one another, forming a closed behavioral cycle that clearly and comprehensively demonstrates the diving process as well as the psychological characteristics and behavioral patterns of the athletes during the diving competitions. At the same time, “pace-setting” is the key to organically connecting various stages throughout the process of PPRs and clearly and comprehensively demonstrating the diving process, psychological characteristics, and behavioral patterns of top athletes during such a process. This research mainly focused on PPRs of individual diving divisions; hence, the constructed model is suitable for individual diving athletes in the divisions of platform and springboard diving.

## Data Availability Statement

The raw data supporting the conclusions of this article will be made available by the authors to any qualified researcher, if we do not utilize it for any other purpose.

## Ethics Statement

The studies involving human participants were reviewed and approved by the Beijing Normal University. The ethics committee waived the requirement of written informed consent for participation.

## Author Contributions

QY designed the study. All authors analyzed the data, wrote and revised the manuscript, contributed to the manuscript revision, and approved the submitted version.

## Conflict of Interest

The authors declare that the research was conducted in the absence of any commercial or financial relationships that could be construed as a potential conflict of interest.
